# Therapy Patterns and Surveillance Measures of Inflammatory Bowel Disease Patients beyond Disease-Related Hospitalization: A Claims-Based Cohort Study

**DOI:** 10.1159/000524741

**Published:** 2022-04-27

**Authors:** Caroline Bähler, Beat Brüngger, Eva Blozik, Stephan R. Vavricka, Alain M. Schoepfer

**Affiliations:** ^a^Department of Health Sciences, Helsana Insurance Group, Zurich, Switzerland; ^b^Center for Gastroenterology and Hepatology, Zurich, Switzerland; ^c^Gastroenterology and Hepatology, University Hospital Zurich, Zurich, Switzerland; ^d^Division of Gastroenterology and Hepatology, Centre Hospitalier Universitaire, Vaudois/CHUV and University of Lausanne, Lausanne, Switzerland

**Keywords:** Inflammatory bowel disease, Surveillance, Healthcare utilization, Biologics

## Abstract

**Introduction:**

Medical care and surveillance of inflammatory bowel disease (IBD) patients have been shown to be far from satisfactory. Data on therapy patterns and surveillance measures in IBD patients are scarce. We, therefore, aimed to compare the therapy patterns and surveillance management of IBD patients in the year before and after IBD-related hospitalization.

**Methods:**

We examined medical therapy, surveillance management (influenza vaccination, dermatologist visits, Pap smear screening, creatinine measurements, iron measurements, and ophthalmologist visits) and healthcare utilization in 214 ulcerative colitis (UC) and 259 Crohn's disease (CD) patients who underwent IBD-related hospitalization from 2012 to 2014.

**Results:**

IBD-related drug classes changed in 64.5% of IBD patients following hospitalization. During the 1-year follow-up period, biological treatment increased in UC and CD patients, while steroid use decreased. Following hospitalization, 63.1% of UC and 27.0% of CD patients received 5-ASA. Only 21.6% of all IBD patients had a flu shot, and 19.6% of immunosuppressed IBD patients were seen by a dermatologist in the follow-up; other surveillance measures were more frequent. Surveillance before hospital admission and consultations by gastroenterologists were strongly correlated with surveillance during the postoperative follow-up, while gender and diagnosis (UC vs. CD) were not. During the 1-year follow-up, 20.5% of all IBD patients had no diagnostic or disease-monitoring procedure.

**Discussion/Conclusion:**

Surveillance measures for IBD patients are underused in Switzerland. Further research is needed to examine the impact of annual screenings and surveillance on patient outcomes.

## Introduction

Ulcerative colitis (UC) and Crohn's disease (CD), the two main subtypes of inflammatory bowel disease (IBD), are chronic relapsing diseases of the gastrointestinal tract. The etiology and pathophysiology of UC and CD are not yet entirely understood. However, a combination of immunological and environmental factors, as well as genetic susceptibility, seems to be responsible for the onset of UC and CD [1, 2, 3, 4, 5, 6]. The prevalence of IBD is estimated to be approximately 0.3–0.6% in Western countries and seems to be increasing worldwide [7, 8, 9, 10, 11, 12, 13].

Due to the complexity of disease, the management of IBD patients is challenging and consists of scheduled follow-up visits and close monitoring of disease activity, as well as several surveillance measures to reduce the risk of flares and long-term complications [14, 15, 16]. Medical therapy is of great importance to prevent disease progression, reduce the risk of flares, and lower the likelihood of other immune-mediated inflammatory diseases in IBD patients. Medications are the main pillar of treatment in patients with UC or CD [17, 18]. Over the course of an IBD-related hospitalization, the medical treatment pattern of patients is typically reevaluated and may undergo several modifications. Of note, considerable differences in treatment practices exist between physicians [19, 20]. In general, a more rapid step-up approach that includes the close monitoring of biomarkers and mucosal disease is currently recommended, especially in hospitalized patients with a more severe disease course [15, 16].

While optimal care of IBD patients is often discussed, in terms of medical and surgical treatment and diagnostic and monitoring procedures, it also requires close attention to surveillance and health maintenance [21]. Surveillance that is strongly recommended by the European Crohn's and Colitis Organization (ECCO) and international guidelines for UC and CD patients includes, among other things, annual influenza vaccinations, annual dermatologist visits in immunosuppressed patients, annual Papanicolaou (Pap) smear screening in immunosuppressed female patients, annual renal function testing (creatinine measurements) in patients receiving 5-aminosalicylic acid (5-ASA), iron-deficiency anemia screening (iron measurements) in immunosuppressed patients, and ophthalmologist visits in patients receiving corticosteroids [15, 16, 22, 23, 24]. On the other hand, surveillance in IBD patients has been identified as far from satisfactory in studies from the USA [25, 26, 27], and preventive health service in IBD appears to even be worse than among primary care patients [28].

The present study, with real-life data from Switzerland, compared the therapy patterns of UC and CD patients during a 1-year period before and a 1-year period after an IBD-related hospitalization, focusing on IBD-related medication. We also examined surveillance management (influenza vaccination, dermatologist visits, Pap smear screening, creatinine measurements, iron measurements, and ophthalmologist visits) as well as diagnostic and monitoring procedures and rehospitalization rates in hospitalized IBD patients. We hypothesized that, based on the step-up approach, a greater proportion of patients would have been treated with immunomodulators, biologics, and/or calcineurin inhibitors, as well as with drug combinations following IBD-related hospitalization. We also hypothesized that surveillance management following hospitalization would be underused in this study population. Only scarce information exists on surveillance management in UC and CD patients [28].

Real-world data obtained from routinely collected sources like health insurance claims are increasingly being used to track treatment patterns among individuals [29]. Such data are reliable and comprehensive, as they cover different aspects of healthcare services across diverse healthcare settings. Consequently, claims data offer a good opportunity to observe the course of treatment patterns and surveillance measures in patients with chronic diseases [30, 31].

## Materials and Methods

### Study Design and Study Population

This retrospective, observational study is based on Helsana claims data of adult patients hospitalized, due to UC or CD, between 2012 and 2014. The Helsana Group is one of the largest health insurers in Switzerland, ensuring approximately 15% of the entire Swiss population across all regions and age groups. The Swiss diagnosis-related group system was introduced in 2012 and refers to ICD-10 codes and applied procedures. As such, our analysis centers on ICD-10 codes pertaining to either CD (K50) or UC (K51) as the primary discharge diagnosis. Patients aged 17 years or younger at the time of their index hospitalization were excluded.

A total of 588 adult patients were identified with at least one IBD-related hospitalization. Altogether, 34 (8.4%) patients who died and 81 (13.8%) patients with missing data (patients without full coverage during the observation time, Helsana employees, and patients seeking asylum in Switzerland) were excluded. Consequently, the final study population comprised 214 (45.2%) UC and 259 (54.8%) CD patients who were hospitalized with a main diagnosis of IBD between January 1, 2012, and December 31, 2014.

### Measures

The change in IBD-related medications prior to and following the index hospitalization was analyzed. IBD-related medications are grouped via Anatomical Therapeutic Chemical codes: 5-ASA (mesalazine and sulfasalazine), steroids (prednisone, budesonide), immunomodulators (azathioprine, 6-mercaptopurine, methotrexate), biologics (TNF antagonists: infliximab, adalimumab, golimumab, certolizumab pegol; and integrin inhibitors: vedolizumab), and calcineurin inhibitors (tacrolimus). Vedolizumab received its first marketing approval in Switzerland in January 2015 and was, therefore, only present for the follow-up analysis. Following the concept of the therapeutic pyramid in IBD patients, we assigned the following levels of drug therapy: Level 1: 5-ASA; Level 2: steroids; Level 3: immunomodulators; Level 4: biologics; and Level 5: calcineurin inhibitors [32, 33]. In addition, the following surveillance measures, all covered by mandatory health insurance, were examined: (1) the proportion of IBD patients with annual influenza vaccinations, (2) the proportion of immunosuppressed IBD patients with annual dermatologist visits, (3) the proportion of immunosuppressed female IBD patients with annual Pap smear screening, (4) the proportion of patients receiving 5-ASA undergoing annual creatinine measurements, (5) the proportion of immunosuppressed IBD patients with iron measurements, and (6) the proportion of patients receiving steroids with annual ophthalmologist visits.

Moreover, resource utilization in the follow-up year was assessed, including the frequency of rehospitalizations (with and without disease-related surgical procedures), the proportion of patients with at least one face-to-face consultation, the median number of consultations (by primary care physicians and gastroenterologists), and the proportion of patients with at least one diagnostic or monitoring procedure. The diagnostic and monitoring procedures consisted of abdominal computed tomography (CT) scanning, abdominal and/or pelvic magnetic resonance imaging (MRI), abdominal sonographies, colonoscopies, and fecal calprotectin measurements. Surgery was defined using DRG Codes (“surgical”); disease-related surgery was defined by additionally using ICD-10 Codes (“K: diseases of the digestive system”).

Patient characteristics (age group: 18–40, 41–60, and 61+ years; gender; additional chronic conditions; surgery vs. no surgery at index hospitalization) and the patient's type of health insurance plan (being in a managed care model or having supplementary hospital insurance) were considered during analysis. In Switzerland, compulsory health insurance coverage of each resident is financed by premiums and consists of deductibles and co-payments. Residents may take out a supplementary hospital insurance policy, or they may choose a managed care model. Managed care models also go along with lower premiums; but they, in turn, restrain the free choice of physicians. Regional factors (type of residence and language region) had no impact upon the surveillance management during preanalyses and were, therefore, not included in the final analysis. Twenty-one additionally treated chronic conditions were identified on the basis of the Anatomical Therapeutic Chemical classification system, using an updated measure of the Pharmacy-based Cost Group (PCG) model: acid-related disorders, bone diseases (osteoporosis), cancer, cardiovascular diseases (including hypertension), dementia, diabetes mellitus, epilepsy, glaucoma, gout/hyperuricemia, HIV, hyperlipidemia, iron deficiency anemia, migraines, pain, Parkinson's disease, psychological disorders (sleep disorders, depression), psychoses, respiratory illness (asthma, COPD), rheumatologic conditions, thyroid disorders, and tuberculosis [34].

### Statistical Analysis

Descriptive statistical analysis was performed to assess for differences between patients with UC and CD by means of the Fisher's exact test for dichotomous variables, Pearson's χ^2^ test for categorical variables, and Wilcoxon rank sum test for continuous variables. Changes in drug classes prior to and after the index hospitalization are presented using alluvial plots [35]. Monthly changes in drug classes are shown by means of transversal state distributions plots [36] and stacked bar charts, both following the concept of the therapeutic pyramid in IBD. We conducted multivariate logistic regression modeling to analyze predictors of surveillance management (taking into account the diagnosis, age group, gender [if appropriate], number of additional chronic diseases, surveillance in the preceding year, consultations by gastroenterologists, managed care, and supplementary hospital insurance) and combined medical treatment in the follow-up (taking into account the diagnosis, age group, gender, number of additional chronic diseases, combined medical treatment in the preceding year, and consultations by primary care physicians and by gastroenterologists). McNemar's χ^2^ test was used to evaluate for differences prior to and following index hospitalization, in terms of medications and healthcare utilization. All analyses were conducted using R, version 3.5.0 (R Foundation for Statistical Computing, Vienna, Austria; www.r-project.org), with a two-tailed *p* ≤ 0.05 set as the criterion for statistical significance.

## Results

### Baseline Characteristics

Characteristics of the study population are summarized in Table 1. Of the 473 patients with a main diagnosis of UC or CD, 53.7% were female. UC patients were, on average, older and had more additionally treated chronic conditions; but they less often underwent surgery during their index hospitalization than CD patients. The surgical procedures in UC patients mainly consisted of ileostomy (*n* = 7), total colectomy (*n* = 5), and subtotal colectomy (*n* = 5), among whom 2 patients had undergone a proctocolectomy. In CD patients, the most frequent surgical procedures were subtotal colectomy (*n* = 54), peritoneal adhesiolysis (*n* = 28), and ileal resection (*n* = 22).

### Drug Prescriptions before and after IBD-Related Hospitalization

The IBD-related drug classes of UC and CD patients changed in 305 (64.5%) of IBD patients following IBD-related hospitalization (65.0% of UC and 64.1% of CD patients). Of all IBD patients, 148 (31.3%) had at least one combination therapy (5-ASA and/or biologics and/or immunomodulators) in the follow-up (35.0% of UC and 28.2% of CD patients). In contrast, combination therapy was present in only 14.5% of UC and 11.6% of CD patients in the year before the index hospitalization. Patients with CD were less likely to receive more than one IBD-related drug compared to UC patients (OR: 0.50, 95% CI: 0.31–0.79, *p* = 0.003). Younger age and combined medical treatment prior to the index hospitalization were positively associated with combined medical treatment during the follow-up. While consultations by primary care physicians did not impact combination therapy, consultations by a gastroenterologist increased the likelihood that a patient received combined medical treatment.

**Fig. 1:**
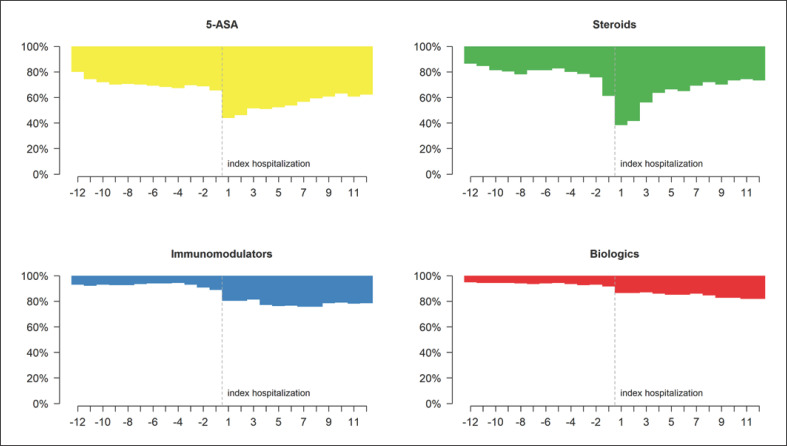
Monthly changes in IBD-related drug classes prior to and post hospitalization in UC patients.

**Fig. 2:**
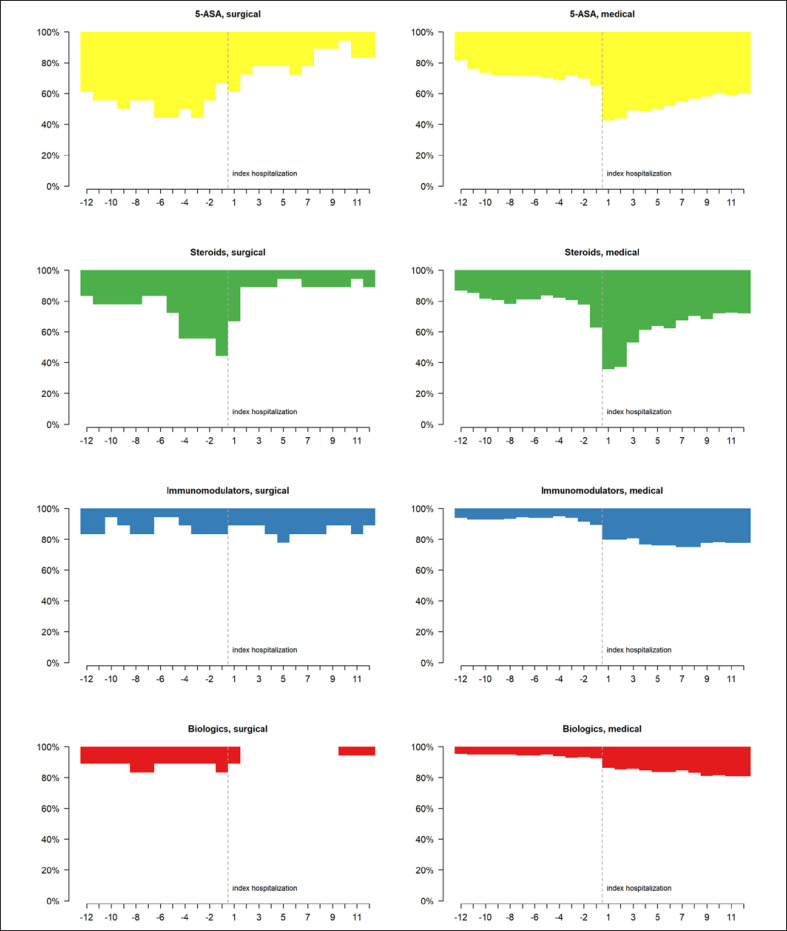
Monthly changes in IBD-related drug classes prior to and post hospitalization in UC patients with (=surgical) and without (=medical) a disease-related surgery at index hospitalization.

The most frequently prescribed IBD-related medications in UC patients were steroids and 5-ASA, both in the year prior to and following IBD-related hospitalization (Fig. 1; online suppl. Table 1; for all online suppl. material, see www.karger.com/doi/10.1159/000524741). Generally, there were a gradual increase in biological prescriptions and a marked decrease in steroid prescriptions over the 12 months of follow-up. This decrease was most prominent in the third month after hospitalization and accompanied by a considerable increase in 5-ASA prescriptions in that same month. Figure 2 depicts the monthly changes in IBD-related drug classes in UC patients with surgical or medical procedures at index hospitalization. In UC patients having undergone surgical treatment, 33.3% stopped treatment with 5-ASA and 44.4% no longer received steroids during the follow-up, while in UC patients with medical treatment, 26.5% started treatment with 5-ASA and 27.6% were newly treated with steroids on the follow-up. Steroid prescriptions, particularly in combination with 5-ASA prescriptions, declined over time in this latter group of patients. Moreover, the number of patients taking immunomodulators or biologics more than doubled after hospitalization, due to the sharp increase in their use in medically treated patients during the index hospitalization. Changes in drug classes − including combinations − prior to and post hospitalization, in UC patients with surgical or medical procedures, are shown in online supplementary Figures 1–3, as well as in online supplementary Tables 2 and 3.

**Fig. 3:**
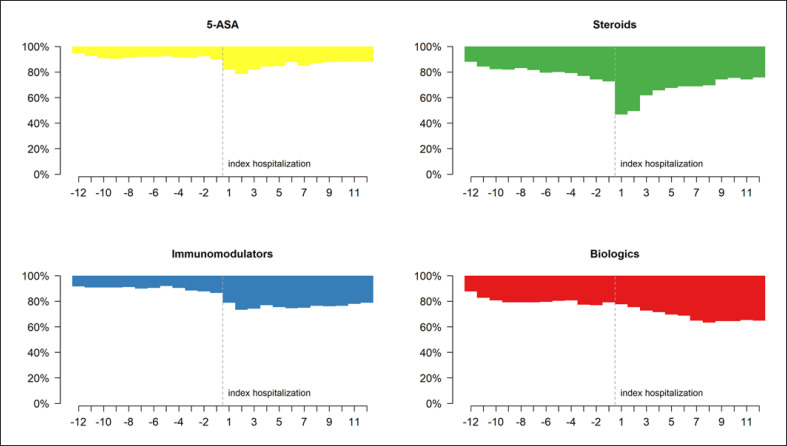
Monthly changes in IBD-related drug classes prior to and post hospitalization in CD patients.

**Fig. 4:**
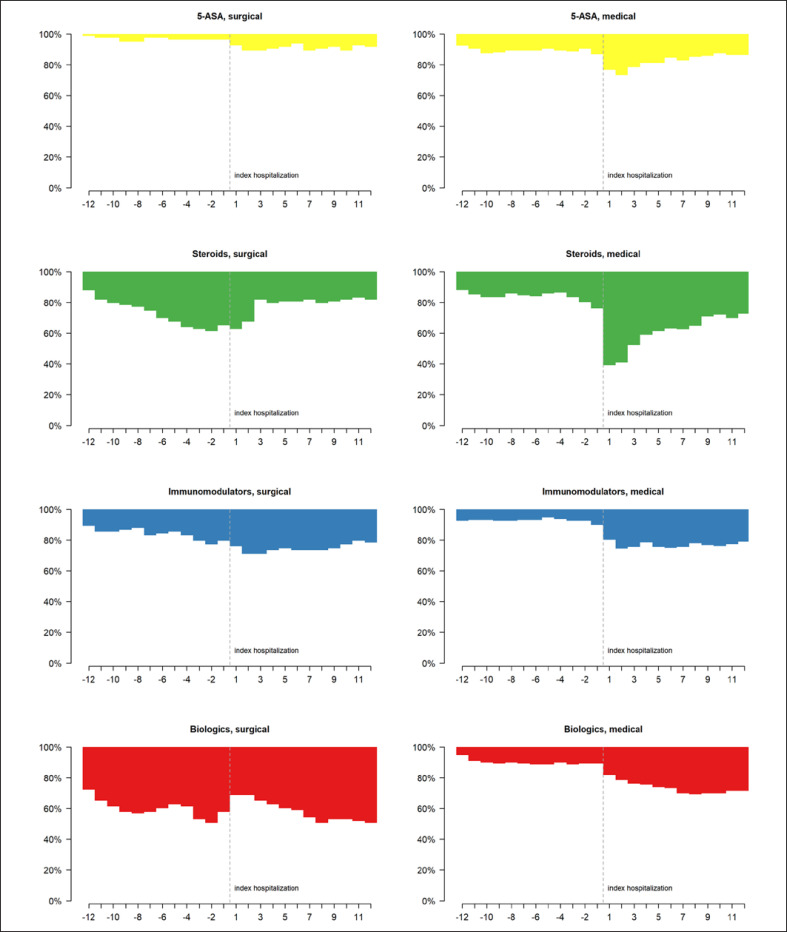
Monthly changes in IBD-related drug classes prior to and post hospitalization in CD patients with (=surgical) and without (=medical) a disease-related surgery at index hospitalization.

CD patients most frequently received steroids, followed by biologics both in the year prior to and following IBD-related hospitalization (Fig. 3; online suppl. Table 4). Within the first 3 months following hospitalization, there was a decrease in steroid prescriptions, especially when combined with 5-ASA. In contrast, biological treatment increased during the 8 months after hospitalization and remained constant thereafter. Figure 4 depicts the monthly changes in IBD-related drug classes in CD patients with surgical or medical procedures at index hospitalization. In CD patients having undergone surgical treatment at index hospitalization, 27.7% stopped treatment with steroids and 8.4% no longer received biologics during the follow-up. Meanwhile, in CD patients with medical treatment, 36.4% started treatment with steroids and 20.5% were newly treated with biologics during the follow-up. The proportion of patients treated with biologics was significantly higher in patients with surgical treatment at index hospitalization. We found a significant increase in patients taking immunomodulators and in those taking 5-ASA after index hospitalization without any surgical treatment. Interestingly, one-fifth of the CD patients with a surgical index hospitalization and almost one-third of patients with a medical index hospitalization were still treated with 5-ASA during the follow-up. Changes in drug classes − including combinations − prior to and post hospitalization, in CD patients with surgical or medical procedures, are shown in online supplementary Figures 4–6, as well as in online supplementary Tables 5 and 6.

### Surveillance Measures

**Fig. 5:**
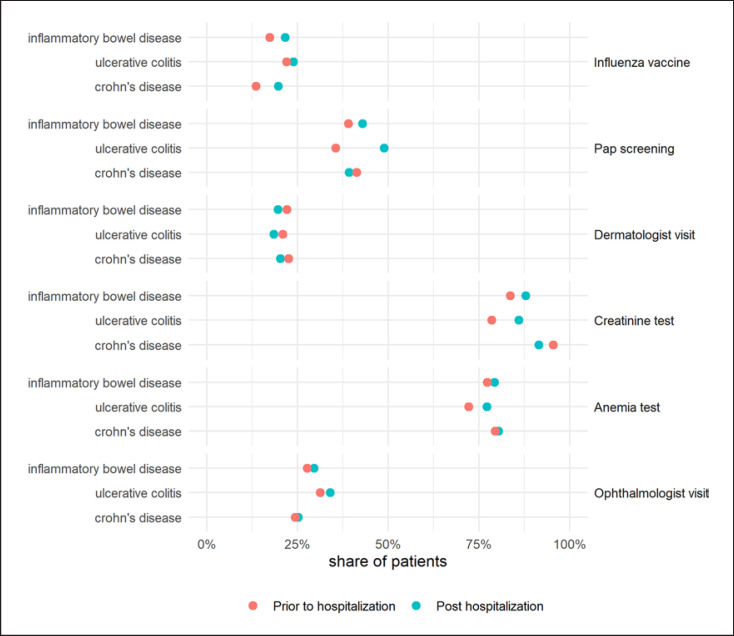
Number of patients undergoing recommended surveillance measures in the year following IBD-related hospitalization.

Surveillance measures in UC and CD patients are listed in Table 2 and depicted in Figure 5. In total, 21.6% (102/473) of all IBD patients had a flu shot in the year following hospitalization, compared to 17.3% (82/473) in the year prior to hospitalization. No statistically significant difference was found between the proportion of vaccinated IBD patients with and without immunosuppression on the follow-up (18.8% vs. 23.3%). Of all IBD patients who were immunosuppressed, 19.6% (49/250) were seen by a dermatologist in the year following hospitalization, compared to 22.1% (32/145) of immunosuppressed patients in the year prior to hospitalization. While 42.9% (51/119) of immunosuppressed female IBD patients underwent Pap smear screening in the following year, this proportion was 39.0% (30/77) in the year prior to the index hospitalization. The proportions of UC and CD patients with annual creatinine measurements, in patients receiving 5-ASA, were 87.8% (180/205) post and 83.6% (122/146) prior to hospitalization. Of all IBD patients who were immunosuppressed, 79.2% (198/250) were screened for iron-deficiency anemia in the year following hospitalization, compared to 77.2% (112/145) of immunosuppressed patients in the year prior to hospitalization. Finally, 29.5% (90/305) of all IBD patients receiving steroids were seen by an ophthalmologist in the year following hospitalization, compared to 27.7% (62/224) patients in the year prior to hospitalization.

Overall, the main predictor of undergoing surveillance measures in the year following hospitalization was the number of consultations with the physician; meanwhile, diagnosis (CD vs. UC), gender, and additional chronic diseases had no impact. Consultations by gastroenterologists were more strongly associated with surveillance than consultations with primary care physicians. Online supplementary Table 7 shows the predictors of each surveillance measure. Older age and having supplementary hospital insurance were both associated with higher odds of having an influenza vaccination on the follow-up (OR: 2.88, 95% CI: 1.39–6.13, *p* = 0.005 and OR: 2.22, 95% CI: 2.16–4.20, *p* = 0.015, respectively). Older age was also associated with higher odds of an ophthalmologist visit during the follow-up (OR: 2.16, 95% CI: 1.04–4.53, *p* = 0.039 for patients aged 41–60 years, and OR: 3.14, 95% CI: 1.46–6.89, *p* = 0.004 for patients aged over 60 years). Patients in a managed care model were less likely to see a dermatologist (OR: 0.39, 95% CI: 0.17–0.86, *p* = 0.022), but more likely to undergo creatinine measurement (OR: 3.59, 95% CI: 1.34–10.69, *p* = 0.015). Surveillance in the year preceding hospitalization was significantly and positively associated with an influenza vaccination, dermatologist visit, iron-deficiency anemia screening, and ophthalmologist visit, but not with Pap smear screening or creatinine measurement during the follow-up. However, these results need to be interpreted with caution due to wide confidence intervals.

### Healthcare Utilization

At least one rehospitalization was identified in about 40% of all IBD patients (online suppl. Table 8). Disease-related surgery was more often performed in CD versus UC patients over the 12 months of follow-up. Ninety-three percent of all UC patients and 97.7% of all CD patients had at least one face-to-face consultation with a primary care physician or gastroenterologist during the follow-up. Ninety-seven (20.5%) of all IBD patients had no diagnostic or monitoring procedure (colonoscopy, abdominal sonography, CT or MRI, or fecal calprotectin) during the 1-year follow-up (online suppl. Table 8). Colonoscopy was performed in almost half of all UC and CD patients during the follow-up, with no significant difference between the two groups. However, fecal calprotectin measurements and abdominal imaging (CT, MRI, sonography) were more frequently used in CD than in UC patients. In total, 49.3% of IBD patients had one or more colonoscopies, while only 32.3% of IBD patients had fecal calprotectin measurements in the year after their index hospitalization.

## Discussion/Conclusion

Our study carries several messages that are clinically relevant. First, IBD-related drug classes changed in almost two-thirds of IBD patients following hospitalization. Some of the changes were to be expected, such as the increase in biologics and the decrease in steroid use, whereas others, like the increase in 5-ASA use, were not. Second, about one-fifth of IBD patients had a flu shot, and as many as 20.5% had no diagnostic or monitoring procedure over 1 year of follow-up, showing that IBD surveillance measures are underused.

A higher proportion of patients were treated with biologics in the year after IBD-related hospitalization relative to the year before, notably after surgical procedures. We assume that these patients had had a more severe disease course prior to hospitalization. While the therapeutic strategy of starting with 5-ASA, steroids, or immunomodulators with escalation to more effective drugs only after treatment failure was common in the past, a more rapid step-up approach based on close monitoring of biomarkers and mucosal disease is now recommended [16]. This recommendation, covering the early administration of biologics, holds true for UC [15] and CD patients [37, 38, 39]. While steroid intake declined in CD patients with a surgical hospitalization, there was an increase in medically treated CD patients. The decrease was to be expected as surgery was able to deal with the mechanical and/or inflammatory complications, thereby making continued steroid treatment no longer necessary. Interestingly, more than one-fourth of all CD patients (one-fifth of CD patients post hospitalization) were treated with 5-ASA, despite weak evidence supporting 5-ASA use in CD patients, especially postoperatively. This finding is, however, in line with previously reported findings [20]. The high proportion of patients not taking any IBD-related medication preceding the index hospitalization could be attributed to the well-documented diagnostic delay in IBD patients [40, 41]. The high proportion of patients who were not taking any IBD-related medication following hospitalization might be because they were asymptomatic thereafter and, therefore, abstained from taking any medications despite physicians' recommendations. Further reasons may be the lack of effectiveness, intolerance to the drugs, or infections. The proportion of UC patients with a total colectomy who theoretically do not need any IBD-related medication is negligible.

Surveillance is strongly recommended for both UC and CD patients to reduce the risk of flares and long-term complications in patients with these challenging diseases [15, 16]. Screenings serve to detect early-related comorbidities like dysplasia/cancer, iron-deficiency anemia, and renal disease. A recent study found a considerably high prevalence of concomitant chronic diseases, like cancer and iron-deficiency anemia, in Swiss IBD patients [42]. However, little information exists regarding the uptake of a diversity of surveillance measures in IBD patients, mainly from the USA. Of the six preventive surveillance measures assessed in the present study, all but one are strongly recommended, while ophthalmologist visits are to be considered. Though, only one-fifth of all IBD patients had a flu shot. Consistent with the present findings, only 28.1% of immunosuppressed IBD patients reported receiving regular flu shots in the USA [26]. Another US study found considerably higher rates of patients with self-reported influenza vaccination, though rates were lower in patients who were not immunosuppressed [27]. Especially in IBD patients who are starting treatment with TNF antagonists, the risk of serious infections (including respiratory tract infections) leading to hospitalization is high [43]. Merely 20% of immunosuppressed IBD patients were seen by a dermatologist in the follow-up in our study. In a recent US cohort study, only 21.3% of IBD patients consulted a dermatologist [25]. However, dermatologic manifestations of IBD are common, and certain IBD medications, like TNF antagonists, as well as the disease itself, may increase the risk of skin cancer [44, 45, 46]. Regular screening with a Pap test is widespread and essential for reducing morbidity and mortality associated with cervical cancer [47], especially for patients with chronic diseases like IBD [48, 49], and for patients receiving combined treatment with steroids and immunomodulators [50]. In our sample, Pap smear screening was undertaken more frequently than other surveillance measures, with about 40% of immunosuppressed female patients tested. The prevalence rates of Pap smears during a 3-year follow-up period among female IBD patients in Northern California were 93% [51] and 90%, according to another study conducted in Chicago and Kentucky [28]. The higher rates observed in these studies, relative to ours, are mainly because they adopted a 3-year study period. In line with our results, women with CD had fewer Pap smears than women with UC [51].

Renal manifestations and complications of IBD, and possible side effects of associated drugs, emphasize the need for annual evaluations of renal function [15, 16, 52]. Treatment with 5-ASA may impair renal function in IBD patients, most commonly within the first 12 months of drug initiation, although the increased risk of renal disease in these patients seems to be slight [53, 54]. Interestingly, the proportion of IBD patients receiving 5-ASA who had annual creatinine measurements was high in our population, despite the comparably lower risk of renal comorbidities. One reason for this frequent testing could be that testing is most often conducted by primary care physicians, likely in combination with other tests. The same might hold true for iron measurements. Because of inadequate dietary intake and malabsorption, iron-deficiency anemia was repeatedly stated as one of the most frequent concomitant diseases in IBD patients, especially in patients receiving immunomodulators [55, 56, 57]. Lastly, extraintestinal manifestations affecting the eye (episcleritis, uveitis) are reported in 2–5% of IBD patients [58, 59]. The fact that this examination is only to be considered − but not strongly recommended − annually in IBD patients receiving steroids might be one reason why ophthalmologist visits were infrequent in the present cohort. Still, evaluation of the eye should be a routine component in the care of these patients, since it can be associated with significant morbidity, including blindness [60].

Surprisingly, more than one-fifth of all IBD patients did not have any diagnostic or monitoring procedure during the 1-year follow-up. Especially in patients with CD, reexaminations are important because the correlation between disease activity and symptoms can be low. About 40% of all hospitalized patients in the present study were rehospitalized in the following year. In a recent review, European hospitalization rates varied between 0.5% in a Hungarian and 35% in a Danish cohort over 1 year of observation [61]. Since we looked solely at patients with an index hospitalization and a presumably more severe disease course, our observed rates were higher than this.

Our study has several strengths and limitations worth mentioning. Its strengths are the highly reliable and comprehensive, population-based data set available for analysis. To the best of our knowledge, this is the first study to evaluate surveillance measures in Swiss IBD patients. Previous research suggests that administrative data, like claims data, are sufficiently accurate to code IBD, though misclassification cannot entirely be ruled out [62]. However, patients with only mild disease might not be hospitalized and, therefore, might not have been captured in the present study. This selection criterion may have resulted in an overestimation of surveillance testing, as these patients may have more physician consultations and, therefore, have a higher likelihood of being informed about surveillance recommendations. In some patients, IBD might have been first diagnosed at the time of the index hospitalization, which is the reason no medical treatment had been initiated in the year before. Unfortunately, this information is lacking. Furthermore, no measures of disease severity or disease duration were available to control for the appropriateness of medical therapy. Further aspects of preventive, monitoring and surveillance measures in IBD patients are just as important for the course of the disease, like behavioral aspects or further screenings and vaccinations. Unfortunately, these measures could not be taken into account by means of our claims data. Nevertheless, all depicted surveillance measures each have a very important impact on morbidity and mortality in IBD patients.

Surveillance and general health maintenance are decisive in patients with a chronic disease, since the disease itself, as well as the drug-related therapy, is often accompanied by preventable adverse effects. Due to the relatively small sample sizes for some of the surveillance measures (e.g., immunosuppressed female patients), further research is needed to clarify the impact of individual and sociodemographic characteristics on the uptake of specific surveillance measures. As per our findings, gastroenterologist visits appear to have a high impact on surveillance and medical treatment. Since most IBD patients have at least one physician consultation per year, the gastroenterologist should also provide guidance to the patient's primary care physician regarding issues like vaccinations, screening, and cancer surveillance [63]. This being said, US gastroenterologists' knowledge about the need for vaccinations in IBD patients is poor, and vaccination is considered to be the responsibility of primary care physicians by the majority of gastroenterologists [64]. Similar results have been observed among Australian gastroenterologists [65]. Further research is needed to learn more about task and responsibility sharing between gastroenterologists and primary care physicians in Switzerland if we are to optimize treatment for patients with IBD.

In conclusion, the IBD-related drug classes changed in almost two-thirds of IBD patients. Treatment with biologics and 5-ASA increased, whereas steroid treatment decreased over the 1-year follow-up. Surveillance and monitoring of IBD patients are far from being satisfactory in Switzerland: only about one-fifth of IBD patients had a flu shot, and as many as 20.5% had no diagnostic or monitoring procedure over the 1-year follow-up. Since most patients have at least one consultation with a primary care physician or gastroenterologist annually, greater awareness among physicians might improve surveillance in these patients and increase the proportion who are treated as recommended by European and international guidelines. Further research is needed to examine the impact of annual screenings and surveillance on patient outcomes.

## Statement of Ethics

The present study falls outside the scope of the Swiss Federal Act on Research involving Human Beings (Human Research Act, HRA) because the study is retrospective and based on anonymized routine administrative claims data. As all requirements of the article 22 of the Swiss data protection law were fulfilled, no patient informed consent and no further ethics approval was needed.

## Conflict of Interest Statement

Stephan R. Vavricka consulted and received speaker's honoraria from Abbvie, UCB, Falk, Janssen, MSD, Tillots, Vifor, Pfizer, Takeda, and Ferring. Alain M. Schoepfer consulted and received speaker's honoraria from Abbvie, UCB, Falk, Janssen, MSD, Tillotts, Vifor, Pfizer, Ferring, Receptos, and Takeda. The other authors have no competing interests to declare.

## Funding Sources

This work was financially supported by MSD Merck Sharp & Dohme AG (Switzerland). The sponsor reviewed, discussed, and approved the protocol CH-LCE-6247/2019-ms-1344. Conduction, analysis, and interpretation of the data, and preparation and submission of the manuscript were solely in the responsibility of the authors, without any obligations to the sponsor.

## Author Contributions

Caroline Bähler, Beat Brüngger, Eva Blozik, Stephan R. Vavricka, and Alain M. Schoepfer assisted in the elaboration of the concept and design of the study. Beat Brüngger made major contributions to the data collection. Caroline Bähler and Beat Brüngger performed the statistical analysis. Caroline Bähler, Beat Brüngger, Eva Blozik, Stephan R. Vavricka, and Alain M. Schoepfer contributed to data interpretation and manuscript writing, and they had full access to all data in the study and had the final responsibility for the decision to submit for publication.

## Data Availability Statement

Individual data cannot be made fully available because the study is based on claims data of the Helsana Group, the owner of the data. Thus, data underlie data protection and privacy restrictions. These restrictions prohibit us from sharing the collected data. However, data can be shared on the reasonable request from PD Dr. Carola Huber (carola.huber@helsana.ch).

## Supplementary Material

Supplementary data

Supplementary data

Supplementary data

Supplementary data

Supplementary data

Supplementary data

Supplementary data

Supplementary data

Supplementary data

Supplementary data

Supplementary data

## Figures and Tables

**Table 1:** Characteristics of the study population (*n* = 473)

Variable	Total *n* = 473	UC 214 (45.2%)	CD 259 (54.8%)	*p* value^a^
Female sex, *n* (%)	254 (53.7)	120 (56.1)	134 (51.7)	ns
Age, years	51.0 (34, 69)	61.0 (40, 74)	46.0 (31, 62)	<0.001
Age groups, *n* (%)				
18–40	163 (34.5)	57 (26.6)	106 (40.9)	<0.001
41–60	134 (28.3)	49 (22.9)	85 (32.8)	
60+	176 (37.2)	108 (50.5)	68 (26.3)	
Chronic conditions (median, IQR)	2.0 (1.0, 4.0)	3.0 (1.0, 5.0)	2.0 (1.0, 4.0)	<0.001
Health insurance plan, *n* (%)				
Managed care	210 (44.4)	99 (46.3)	111 (42.9)	ns
Supplementary hospital insurance	75 (15.9)	36 (16.8)	39 (15.1)	ns
Surgery at index hospitalization, *n* (%)	101 (21.4)	18 (8.4)	83 (32.0)	<0.001

ns, not significant.

a *p* values, assigning the differences between UC and CD patients, were calculated using Fisher's exact test for dichotomous variables, Wilcoxon rank sum test for continuous variables, and χ^2^ test for categorical variables.

**Table 2:** UC and CD patients with surveillance management prior to and following IBD-related hospitalization

	Prior to hospitalization, *n/N* (%)	Post hospitalization, *n/N* (%)
Influenza vaccine		
UC patients	47/214 (22.0)	51/214 (23.8)
CD patients	35/259 (13.5)	51/259 (19.7)
Pap smear screening		
Female UC patients, immunosuppressed	11/31 (35.5)	22/45 (48.9)
Female CD patients, immunosuppressed	19/46 (41.3)	29/74 (39.2)
Dermatologist visit		
UC patients, immunosuppressed	9/43 (20.9)	17/92 (18.5)
CD patients, immunosuppressed	23/102 (22.5)	32/158 (20.3)
Creatinine test		
UC patients taking 5-ASA	80/102 (78.4)	116/135 (85.9)
CD patients taking 5-ASA	42/44 (95.5)	64/70 (91.4)
Iron-deficiency anemia screening		
UC patients, immunosuppressed	31/43 (72.1)	71/92 (77.2)
CD patients, immunosuppressed	81/102 (79.4)	127/158 (80.4)
Ophthalmologist visit		
UC patients taking steroids	34/109 (31.2)	51/150 (34.0)
CD patients taking steroids	28/115 (24.3)	39/155 (25.2)

## Notes

Caroline Bähler and Beat Brüngger share the first authorship.

Stephan R. Vavricka and Alain M. Schoepfer share the last authorship.
