# Supplementary material for: Therapy Patterns and Surveillance Measures of Inflammatory Bowel Disease Patients beyond Disease-Related Hospitalization: A Claims-Based Cohort Study
Source: Inflamm Intest Dis. 2022 Apr 27;7(2):104–17. doi: 10.1159/000524741 (PMC9294938; doi:10.1159/000524741)
Supplement: Supplementary file 1 — Supplementary data [file iid-0007-0104-s01.docx]

**Supplementary Table 7: Multiple logistic regression analyses analyzing the predictors of surveillance management**

| **Surveillance measures**  Odds ratio (95% CI), p-value | | | | | | |
| --- | --- | --- | --- | --- | --- | --- |
| Characteristics | Influenza vaccination | Dermatologist visit | PAP screening | Creatinine measurement | Iron deficiency anemia screening | Ophthalmologist visit |
| CD (versus UC) | 1.307 (0.764-2.265), 0.333 | 0.993 (0.453-2.224), 0.987 | 0.558 (0.236-1.287), 0.175 | 1.975 (0.709-6.238), 0.214 | 1.293 (0.461-3.504), 0.616 | 0.685 (0.396-1.180), 0.173 |
| Age 41-60 years | 0.785 (0.354-1.706), 0.544 | 0.530 (0.206-1.288), 0.171 | 1.344 (0.533-3.432), 0.532 | 0.392 (0.113-1.266), 0.124 | 1.454 (0.485-4.672), 0.512 | **2.161 (1.044-4.531), 0.039** |
| Age 60+ years | **2.884 (1.389-6.132), 0.005** | 0.618 (0.190-1.863), 0.405 | 0.568 (0.170-1.844), 0.349 | 0.872 (0.216-3.509), 0.845 | 1.162 (0.310-4.448), 0.824 | **3.138 (1.455-6.885), 0.004** |
| Female sex | 0.949 (0.549-1.637), 0.850 | 1.315 (0.613-2.852), 0.482 |  | 0.763 (0.289-1.963), 0.576 | 1.178 (0.457-3.054), 0.733 | 1.291 (0.738-2.263), 0.370 |
| Prior surveillance management | **8.993 (4.920-16.845), <0.001** | **14.922 (6.766-34.888), <0.001** | 0.954 (0.420-2.137), 0.909 | 1.419 (0.502-3.854), 0.497 | **7.719 (2.954-21.506), <0.001** | **7.517 (4.046-14.390), <0.001** |
| Consultations by gastroenterologists | **1.030 (1.010-1.052), 0.004** | 1.007 (0.983-1.032), 0.583 | **1.041 (1.010-1.083), 0.023** | **1.066 (1.010-1.139), 0.037** | 1.006 (0.971-1.050), 0.765 | 1.005 (0.986-1.025), 0.630 |
| Additional chronic conditions | 0.990 (0.866-1.129), 0.882 | 0.963 (0.770-1.195), 0.736 | 0.924 (0.739-1.152), 0.482 | 1.208 (0.936-1.598), 0.162 | 0.885 (0.685-1.148), 0.349 | 0.880 (0.759-1.012), 0.080 |
| Managed care | 0.939 (0.546-1.609), 0.818 | **0.390 (0.168-0.858), 0.022** | 0.819 (0.353-1.881), 0.638 | **3.586 (1.341-10.685), 0.015** | 1.090 (0.422-2.853), 0.859 | 1.567 (0.907-2.732), 0.109 |
| Supplementary hospital insurance | **2.222 (1.161-4.204), 0.015** | 1.875 (0.639-5.184), 0.235 | 0.678 (0.173-2.331), 0.550 | 1.425 (0.446-5.677), 0.577 | 0.583 (0.163-2.293), 0.415 | 0.816 (0.384-1.670), 0.587 |
