# Supplementary material for: Therapy Patterns and Surveillance Measures of Inflammatory Bowel Disease Patients beyond Disease-Related Hospitalization: A Claims-Based Cohort Study
Source: Inflamm Intest Dis. 2022 Apr 27;7(2):104–17. doi: 10.1159/000524741 (PMC9294938; doi:10.1159/000524741)
Supplement: Supplementary file 2 — Supplementary data [file iid-0007-0104-s02.docx]

**SUPPLEMENTARY FIGURE 1:** Changes in IBD-related drug classes (including combinations) prior to and post hospitalization in UC patients. Abbreviations: Bio = biologics, IM = immunomodulators, Ster = steroids.

**SUPPLEMENTARY FIGURE 2:** Monthly changes in IBD-related drug classes (including combinations) prior to and post hospitalization in UC patients. Abbreviations: Bio = biologics, IM = immunomodulators, Ster = steroids.

**SUPPLEMENTARY FIGURE 3:** Monthly changes in IBD-related drug classes (including combinations) prior to and post hospitalization in UC patients with (above) and without (below) a disease-related surgery at index hospitalization. Abbreviations: Bio = biologics, IM = immunomodulators, Ster = steroids.

**SUPPLEMENTARY FIGURE 4:** Changes in IBD-related drug classes (including combinations) prior to and post hospitalization in CD patients. Abbreviations: Bio = biologics, IM = immunomodulators, Ster = steroids.

**SUPPLEMENTARY FIGURE 5:** Monthly changes in IBD-related drug classes (including combinations) prior to and post hospitalization in CD patients. Abbreviations: Bio = biologics, IM = immunomodulators, Ster = steroids.

**SUPPLEMENTARY FIGURE 6:** Monthly changes in IBD-related drug classes (including combinations) prior to and post hospitalization in CD patients with (above) and without (below) a disease-related surgery at index hospitalization. Abbreviations: Bio = biologics, IM = immunomodulators, Ster = steroids.
