# Supplementary figures and images for: Therapy Patterns and Surveillance Measures of Inflammatory Bowel Disease Patients beyond Disease-Related Hospitalization: A Claims-Based Cohort Study
Source: Inflamm Intest Dis. 2022 Apr 27;7(2):104–17. doi: 10.1159/000524741 (PMC9294938; doi:10.1159/000524741)

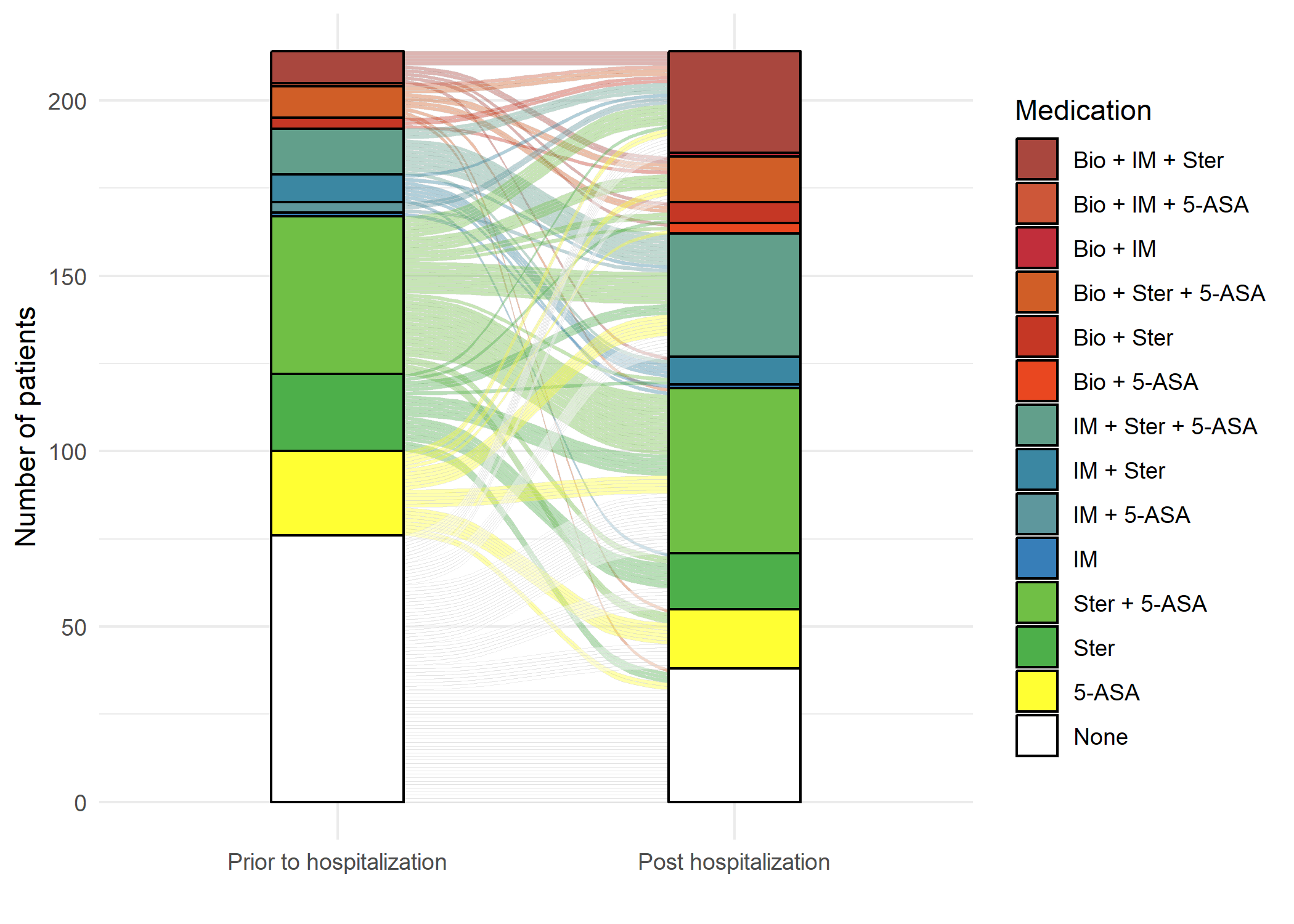

Supplement: Supplementary file 3 — Supplementary data [file iid-0007-0104-s03.tiff]

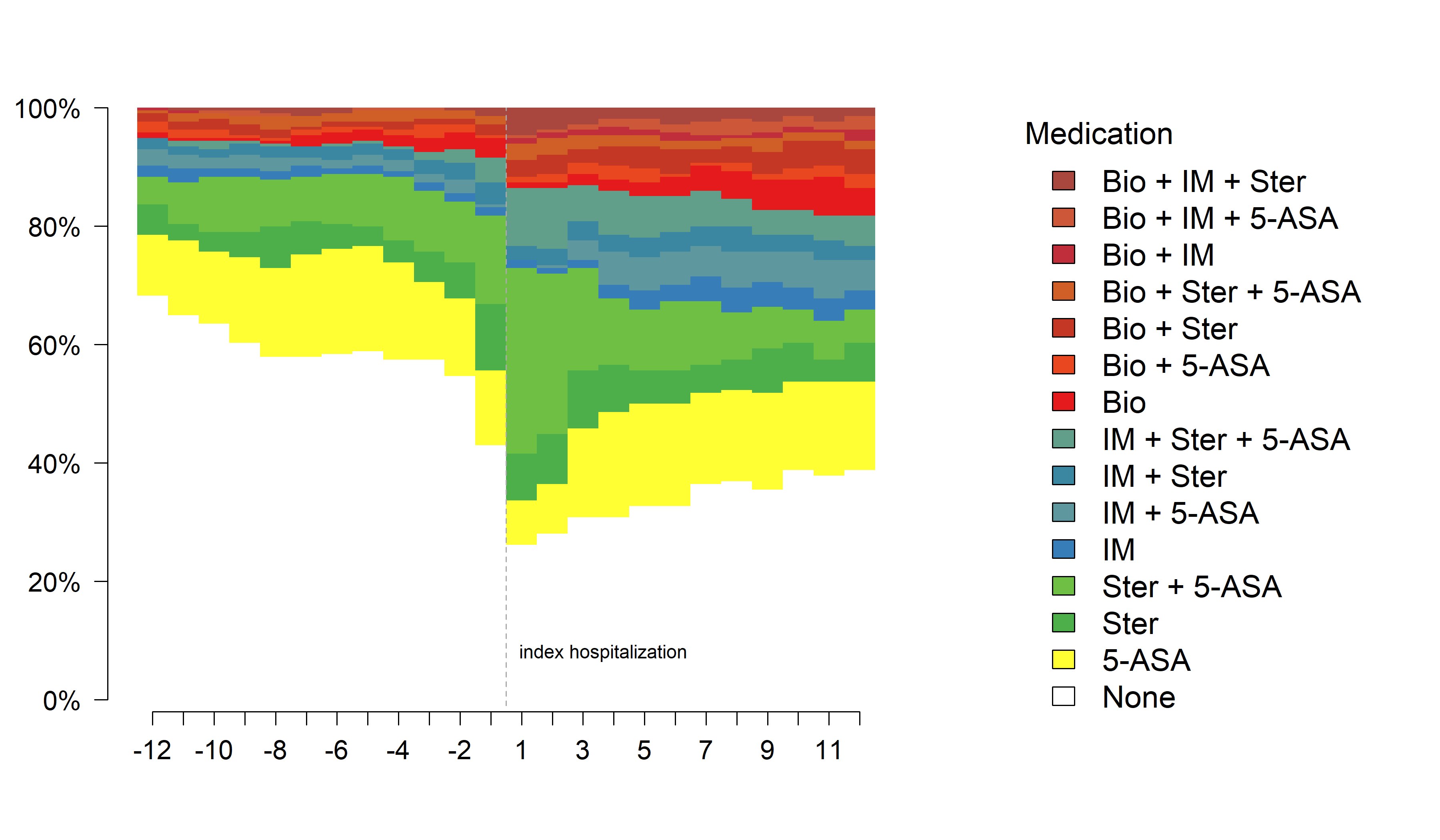

Supplement: Supplementary file 4 — Supplementary data [file iid-0007-0104-s04.tiff]

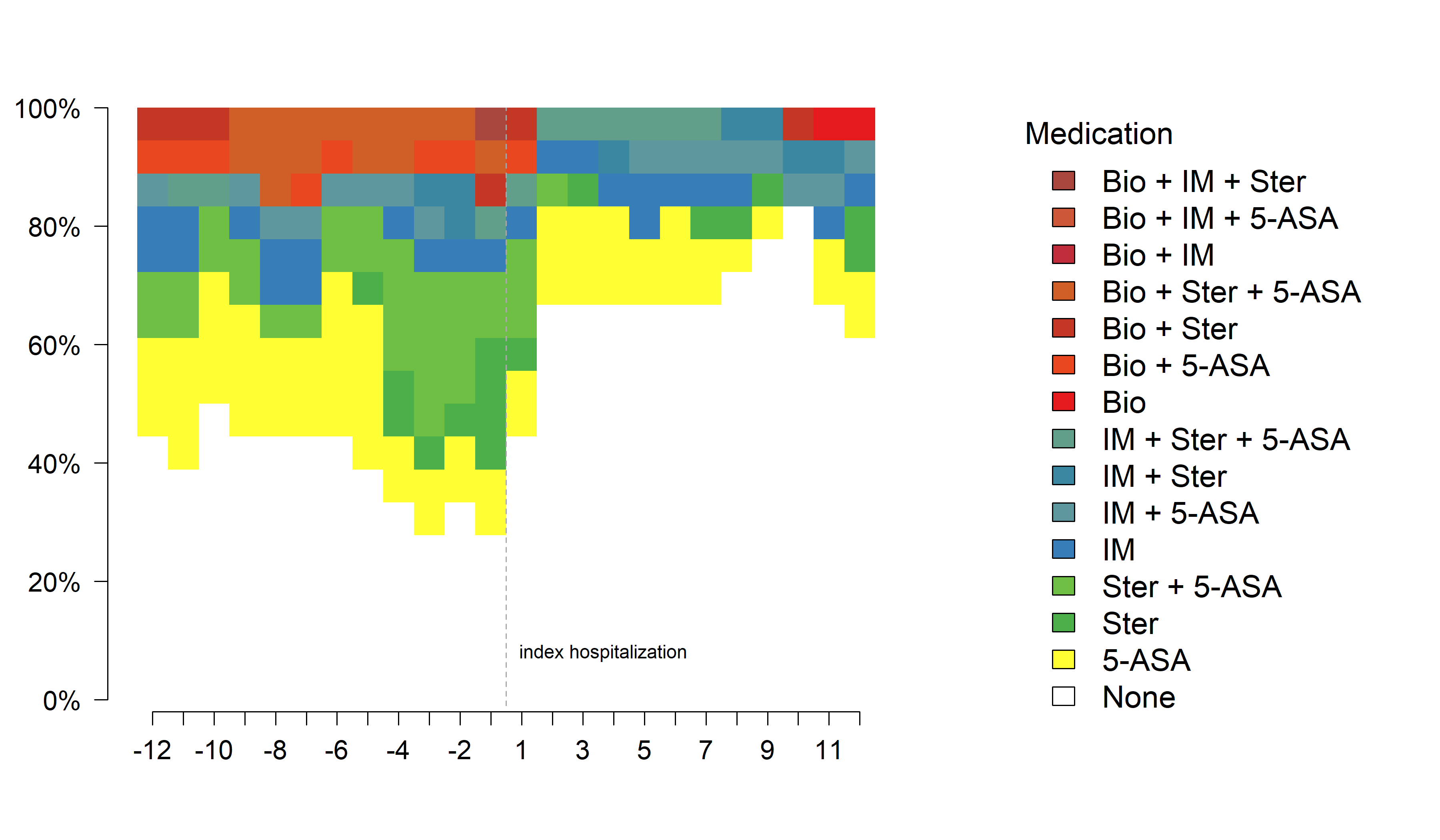

Supplement: Supplementary file 5 — Supplementary data [file iid-0007-0104-s05.tiff]

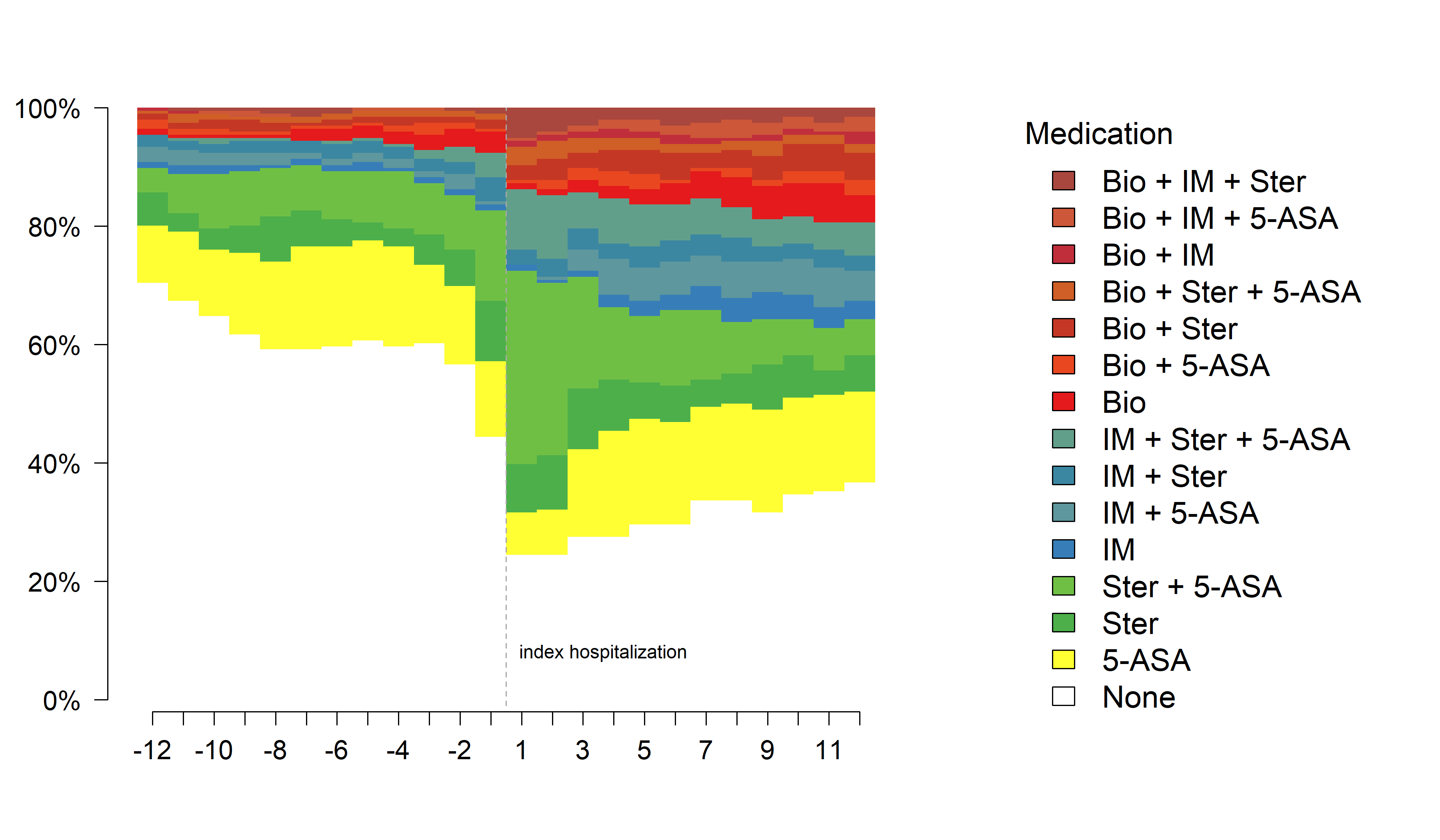

Supplement: Supplementary file 6 — Supplementary data [file iid-0007-0104-s06.tiff]

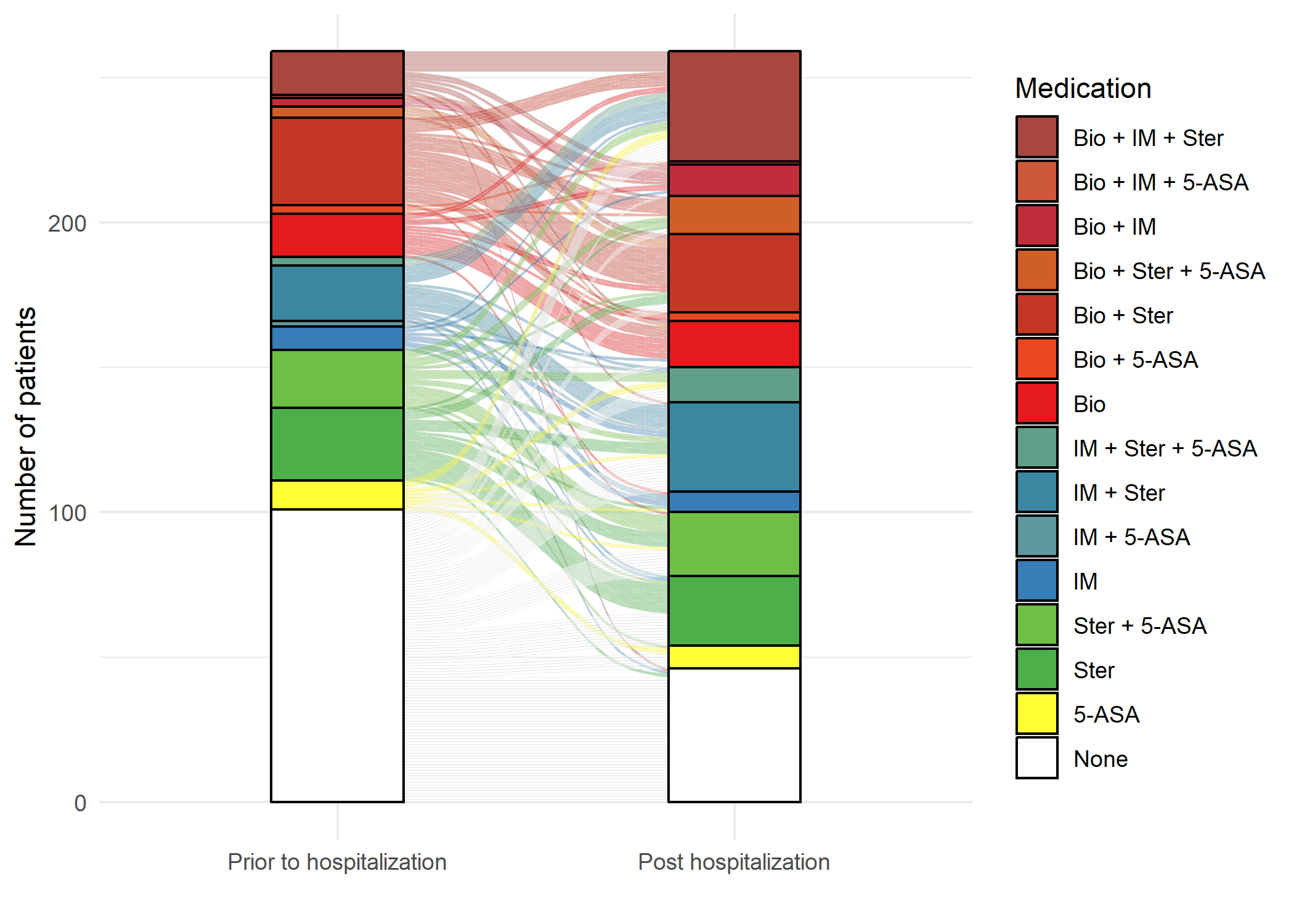

Supplement: Supplementary file 7 — Supplementary data [file iid-0007-0104-s07.tiff]

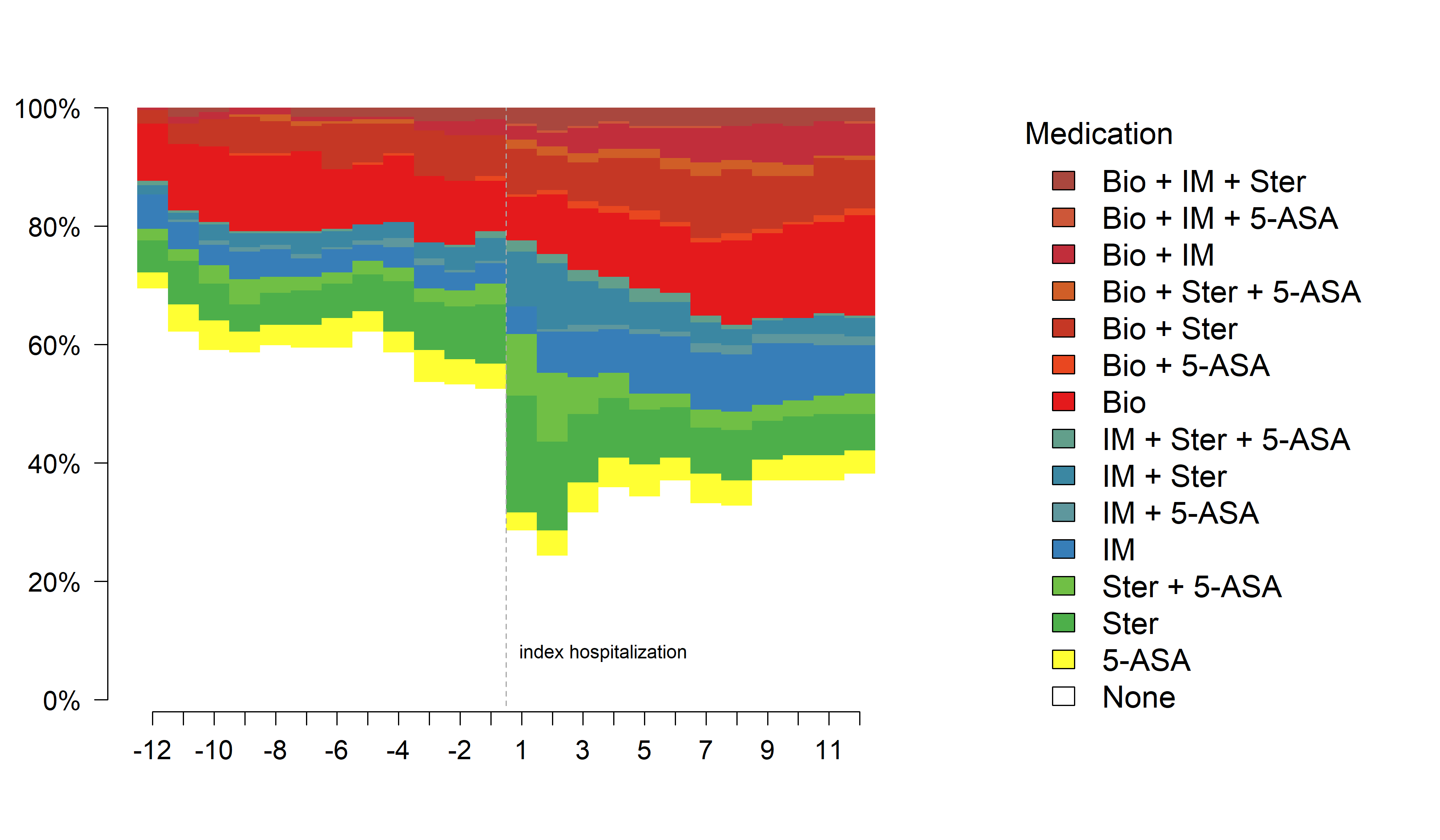

Supplement: Supplementary file 8 — Supplementary data [file iid-0007-0104-s08.tiff]

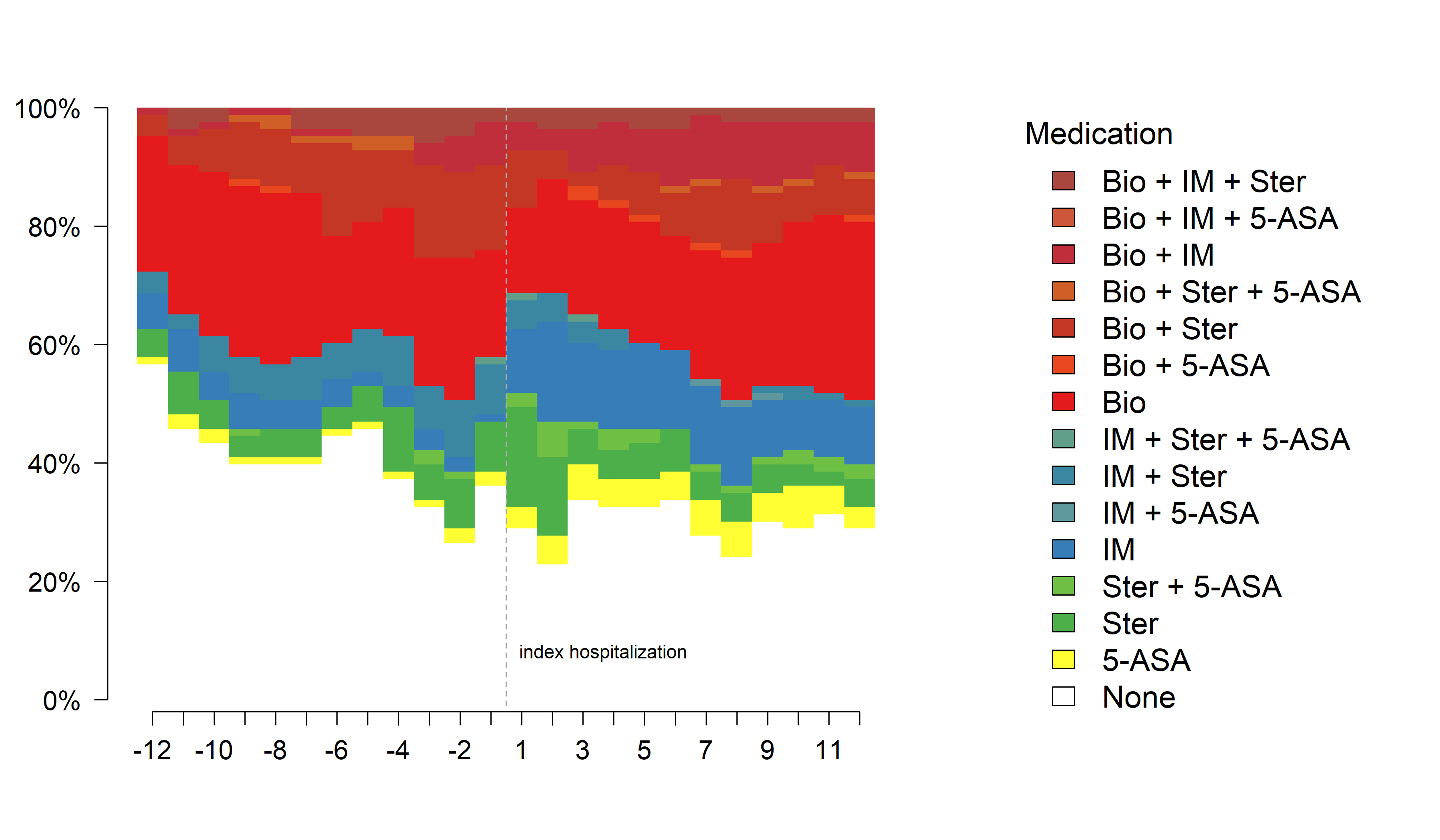

Supplement: Supplementary file 9 — Supplementary data [file iid-0007-0104-s09.tiff]

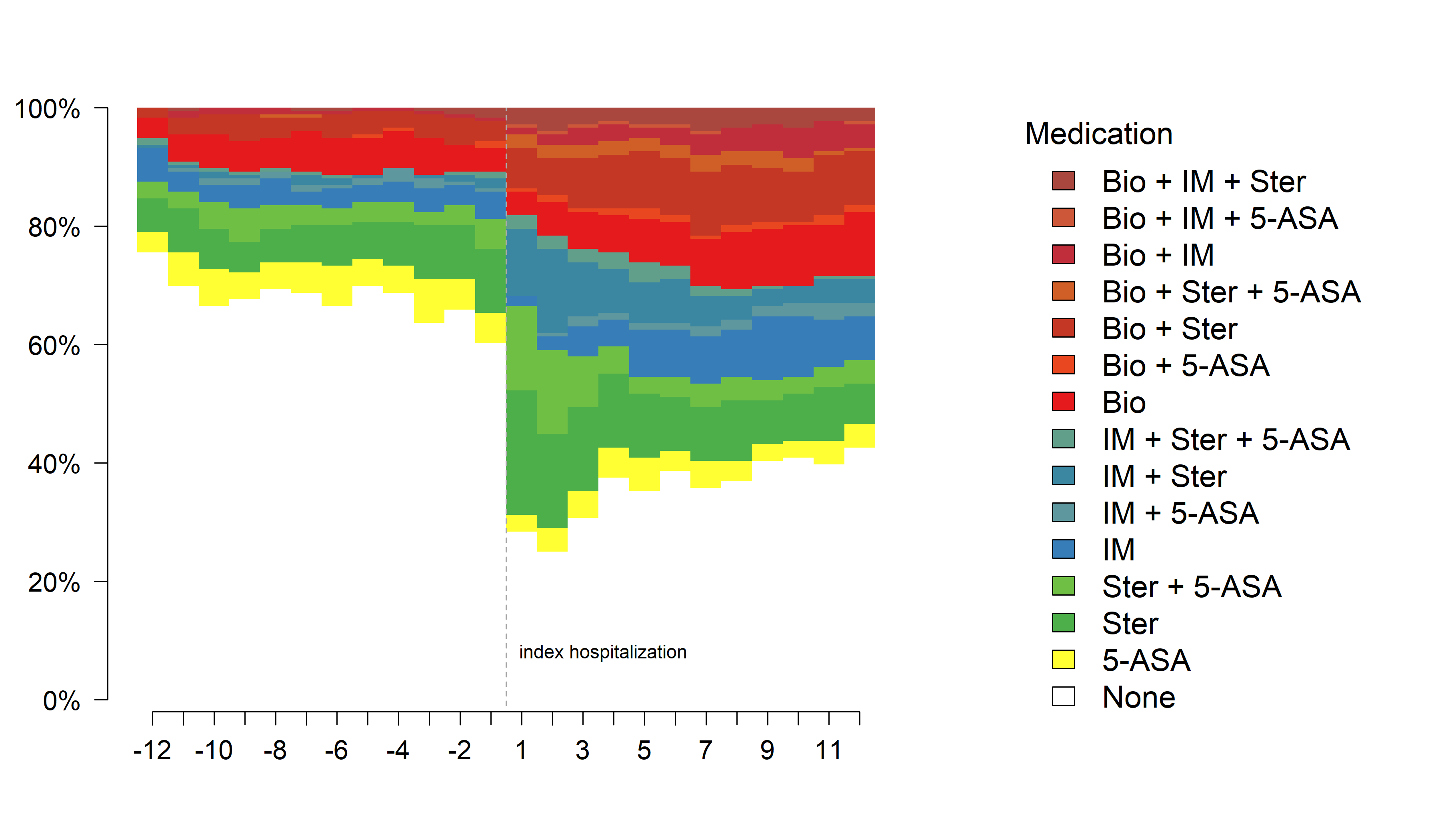

Supplement: Supplementary file 10 — Supplementary data [file iid-0007-0104-s10.tiff]
