# Supplementary material for: Therapy Patterns and Surveillance Measures of Inflammatory Bowel Disease Patients beyond Disease-Related Hospitalization: A Claims-Based Cohort Study
Source: Inflamm Intest Dis. 2022 Apr 27;7(2):104–17. doi: 10.1159/000524741 (PMC9294938; doi:10.1159/000524741)
Supplement: Supplementary file 11 — Supplementary data [file iid-0007-0104-s11.docx]

**Supplementary** **Tables 1-6 and 8**

**Supplementary Table 1: IBD-related medications (including combinations) in UC patients prior to and following index hospitalization (n=214).**

| **Medications in UC patients** | **Prior to hospitalization** | **Post**  **hospitalization** | ***p^a^*** |
| --- | --- | --- | --- |
| No IBD-related drug | 76 (35.5%) | 41 (19.2%) | <0.001 |
| 5-ASA | 102 (47.7%) | 135 (63.1%) | <0.001 |
| Steroids | 109 (50.9%) | 150 (70.1%) | <0.001 |
| Immunomodulators | 35 (16.4%) | 72 (33.6%) | <0.001 |
| TNF Antagonists (TNF) | 18 (8.4%) | 46 (21.5%) | <0.001 |
| Integrin inhibitors (INT, vedolizumab) | 0 | 1 (0.5%) | na |
| Biologics (TNF + INT) | 18 (8.4%) | 46 (21.5%) | <0.001 |
| Calcineurin inhibitors | 5 (2.3%) | 7 (3.3%) | na |
| 5-ASA + Immunomodulators | 24 (11.2%) | 57 (26.6%) | <0.001 |
| 5-ASA + Biologics | 13 (6.1%) | 36 (16.8%) | <0.001 |
| Immunomodulators + Biologics | 10 (4.7%) | 26 (12.1%) | 0.005 |
| Immunomodulators + Biologics + 5-ASA | 8 (3.7%) | 22 (10.3%) | 0.008 |
| 5-ASA + Steroids | 74 (34.6%) | 114 (53.3%) | <0.001 |
| Immunomodulators + Steroids | 30 (14.0%) | 70 (32.7%) | <0.001 |
| Biologics + Steroids | 17 (7.9%) | 44 (20.6%) | na |
| Immunomodulators + Biologics + Steroids | 9 (4.2%) | 26 (12.1%) | na |

^a^ p-values, assigning the differences between prior to and following hospitalization, were calculated using McNemar’s chi-square test; na = not applicable.

**Supplementary Table 2: IBD-related medications (including combinations) in UC patients with an index surgery, prior to and following index hospitalization (n=18).**

| **Medications in UC patients**  **with an index surgery** | **Prior to hospitalization** | **Post**  **hospitalization** | ***p^a^*** |
| --- | --- | --- | --- |
| No IBD-related drug | 4 (22.2%) | 8 (44.4%) | na |
| 5-ASA | 11 (61.1%) | 7 (38.9%) | na |
| Steroids | 11 (61.1%) | 4 (22.2%) | na |
| Immunomodulators | 4 (22.2%) | 4 (22.2%) | na |
| TNF Antagonists (TNF) | 2 (11.1%) | 1 (5.6%) | na |
| Integrin inhibitors (INT, vedolizumab) | 0 | 0 | na |
| Biologics (TNF + INT) | 2 (11.1%) | 1 (5.6%) | na |
| Calcineurin inhibitors | 2 (11.1%) | 0 | na |
| 5-ASA + Immunomodulators | 2 (11.1%) | 2 (11.1%) | na |
| 5-ASA + Biologics | 2 (11.1%) | 1 (5.6%) | na |
| Immunomodulators + Biologics | 1 (5.6%) | 1 (5.6%) | na |
| Immunomodulators + Biologics + 5-ASA | 1 (5.6%) | 1 (5.6%) | na |
| 5-ASA + Steroids | 9 (50.0%) | 2 (11.1%) | na |
| Immunomodulators + Steroids | 3 (16.7%) | 3 (16.7%) | na |
| Biologics + Steroids | 2 (11.1%) | 1 (5.6%) | na |
| Immunomodulators + Biologics + Steroids | 1 (5.6%) | 1 (5.6%) | na |

^a^ p-values, assigning the differences between prior to and following hospitalization, were calculated using McNemar’s chi-square test; na = not applicable.

**Supplementary Table 3: IBD-related medications (including combinations) in UC patients without an index surgery, prior to and following index hospitalization (n=196).**

| **Medications in UC patients**  **without an index surgery** | **Prior to hospitalization** | **Post**  **hospitalization** | ***p^a^*** |
| --- | --- | --- | --- |
| No IBD-related drug | 72 (36.7%) | 33 (16.8%) | <0.001 |
| 5-ASA | 91 (46.4%) | 128 (65.3%) | <0.001 |
| Steroids | 98 (50.0%) | 146 (74.5%) | <0.001 |
| Immunomodulators | 31 (15.8%) | 68 (34.7%) | <0.001 |
| TNF Antagonists (TNF) | 16 (8.2%) | 45 (23.0%) | <0.001 |
| Integrin inhibitors (INT, vedolizumab) | 0 | 1 (0.5%) | na |
| Biologics (TNF + INT) | 16 (8.2%) | 45 (23.0%) | <0.001 |
| Calcineurin inhibitors | 3 (1.5%) | 7 (3.6%) | na |
| 5-ASA + Immunomodulators | 22 (11.2%) | 55 (28.1%) | <0.001 |
| 5-ASA + Biologics | 11 (5.6%) | 35 (17.9%) | <0.001 |
| Immunomodulators + Biologics | 9 (4.6%) | 25 (12.8%) | 0.003 |
| Immunomodulators + Biologics + 5-ASA | 7 (3.6%) | 21 (10.7%) | 0.006 |
| 5-ASA + Steroids | 65 (33.2%) | 112 (57.1%) | <0.001 |
| Immunomodulators + Steroids | 27 (13.8%) | 67 (34.2%) | <0.001 |
| Biologics + Steroids | 15 (7.7%) | 43 (21.9%) | na |
| Immunomodulators + Biologics + Steroids | 8 (4.1%) | 25 (12.8%) | na |

^a^ p-values, assigning the differences between prior to and following hospitalization, were calculated using McNemar’s chi-square test; na = not applicable.

**Supplementary Table 4: IBD-related medications (including combinations) in CD patients prior to and following index hospitalization (n=259).**

| **Medications in CD patients** | **Prior to hospitalization** | **Post**  **hospitalization** | ***p^a^*** |
| --- | --- | --- | --- |
| No IBD-related drug | 102 (39.4%) | 48 (18.5%) | <0.001 |
| 5-ASA | 44 (17.0%) | 70 (27.0%) | 0.001 |
| Steroids | 115 (44.4%) | 155 (59.8%) | <0.001 |
| Immunomodulators | 51 (19.7%) | 99 (38.2%) | <0.001 |
| TNF Antagonists (TNF) | 70 (27.0%) | 104 (40.2%) | <0.001 |
| Integrin inhibitors (INT, vedolizumab) | 0 | 2 (0.8%) | na |
| Biologics (TNF + INT) | 70 (27.0%) | 106 (40.9%) | <0.001 |
| Calcineurin inhibitors | 3 (1.2%) | 3 (1.2%) | na |
| 5-ASA + Immunomodulators | 7 (2.7%) | 24 (9.3%) | <0.001 |
| 5-ASA + Biologics | 8 (3.1%) | 28 (10.8%) | <0.001 |
| Immunomodulators+ Biologics | 19 (7.3%) | 47 (18.1%) | <0.001 |
| Immunomodulators + Biologics + 5-ASA | 2 (0.8%) | 13 (5.0%) | na |
| 5-ASA + Steroids | 28 (10.8%) | 56 (21.6%) | <0.001 |
| Immunomodulators + Steroids | 37 (14.3%) | 76 (29.3%) | <0.001 |
| Biologics + Steroids | 49 (18.9%) | 70 (27.0%) | 0.008 |
| Immunomodulators + Biologics + Steroids | 15 (5.8%) | 35 (13.5%) | na |

^a^ p-values, assigning the differences between prior to and following hospitalization, were calculated using McNemar’s chi-square test; na = not applicable.

**Supplementary Table 5: IBD-related medications (including combinations) in CD patients with an index surgery, prior to and following index hospitalization (n=83).**

| **Medications in CD patients**  **with an index surgery** | **Prior to hospitalization** | **Post**  **hospitalization** | ***p^a^*** |
| --- | --- | --- | --- |
| No IBD-related drug | 16 (19.3%) | 12 (14.5%) | ns |
| 5-ASA | 9 (10.8%) | 17 (20.5%) | ns |
| Steroids | 49 (59.0%) | 31 (37.3%) | 0.001 |
| Immunomodulators | 24 (28.9%) | 30 (36.1%) | ns |
| TNF Antagonists (TNF) | 45 (54.2%) | 44 (53.0%) | ns |
| Integrin inhibitors (INT, vedolizumab) | 0 | 2 (2.4%) | na |
| Biologics (TNF + INT) | 45 (54.2%) | 46 (55.4%) | ns |
| Calcineurin inhibitors | 2 (2.4%) | 2 (2.4%) | na |
| 5-ASA + Immunomodulators | 2 (2.4%) | 2 (2.4%) | na |
| 5-ASA + Biologics | 5 (6.0%) | 9 (10.8%) | ns |
| Immunomodulators + Biologics | 12 (14.5%) | 16 (19.3%) | ns |
| Immunomodulators + Biologics + 5-ASA | 1 (1.2%) | 1 (1.2%) | na |
| 5-ASA + Steroids | 6 (7.2%) | 10 (12.0%) | na |
| Immunomodulators + Steroids | 20 (24.1%) | 12 (14.5%) | na |
| Biologics + Steroids | 30 (36.1%) | 18 (21.7%) | 0.014 |
| Immunomodulators + Biologics + Steroids | 9 (10.8%) | 7 (8.4%) | na |

^a^ p-values, assigning the differences between prior to and following hospitalization, were calculated using McNemar’s chi-square test; na = not applicable; ns = not significant.

**Supplementary Table 6: IBD-related medications (including combinations) in CD patients without an index surgery, prior to and following index hospitalization (n=176).**

| **Medications in CD patients**  **without an index surgery** | **Prior to hospitalization** | **Post**  **hospitalization** | ***p^a^*** |
| --- | --- | --- | --- |
| No IBD-related drug | 86 (48.9%) | 36 (20.5%) | <0.001 |
| 5-ASA | 35 (19.9%) | 53 (30.1%) | 0.007 |
| Steroids | 66 (37.5%) | 124 (70.5%) | <0.001 |
| Immunomodulators | 27 (15.3%) | 69 (39.2%) | <0.001 |
| TNF Antagonists (TNF) | 25 (14.2%) | 60 (34.1%) | <0.001 |
| Integrin inhibitors (INT, vedolizumab) | 0 | 0 | na |
| Biologics (TNF + INT) | 25 (14.2%) | 60 (34.1%) | <0.001 |
| Calcineurin inhibitors | 1 (0.6%) | 1 (0.6%) | na |
| 5-ASA + Immunomodulators | 5 (2.8%) | 22 (12.5%) | <0.001 |
| 5-ASA + Biologics | 3 (1.7%) | 19 (10.8%) | <0.001 |
| Immunomodulators + Biologics | 7 (4.0%) | 31 (17.6%) | <0.001 |
| Immunomodulators + Biologics + 5-ASA | 1 (0.6%) | 12 (6.8%) | 0.003 |
| 5-ASA + Steroids | 22 (12.5%) | 46 (26.1%) | <0.001 |
| Immunomodulators + Steroids | 17 (9.7%) | 64 (36.4%) | <0.001 |
| Biologics + Steroids | 19 (10.8%) | 52 (29.5%) | <0.001 |
| Immunomodulators + Biologics + Steroids | 6 (3.4%) | 28 (15.9%) | na |

^a^ p-values, assigning the differences between prior to and following hospitalization, were calculated using McNemar’s chi-square test; na = not applicable.

**Supplementary Table 8: Healthcare utilization in UC and CD patients during 12-months follow-up.**

|  | **Total** | **UC** | **CD** | ***p^a^*** |
| --- | --- | --- | --- | --- |
|  | 473 | 214 (45.2%) | 259 (54.8%) |  |
| Re-hospitalization | 190 (40.2%) | 84 (39.3%) | 106 (40.9%) | ns |
| Disease-related surgery^b^ | 60 (31.6%) | 16 (19.0%) | 44 (41.5%) | <0.001 |
| Face-to-face consultation | 452 (95.6%) | 199 (93.0%) | 253 (97.7%) | 0.023 |
| Number of consultations^c^ | 23.0 (15, 35) | 23.5 (15, 36) | 22.0 (14, 34) | ns |
| by primary care physicians^c^ | 8.0 (2, 14) | 9.0 (3, 14) | 7.0 (2, 13.5) | 0.043 |
| by gastroenterologists^c^ | 9.0 (4, 17) | 9.0 (3, 15) | 10.0 (4, 18.5) | ns |
| Diagnostic procedure^d^ | 376 (79.5%) | 156 (72.9%) | 220 (84.9%) | 0.001 |
| Abdominal computed tomography scanning (CT) | 79 (16.7%) | 31 (14.5%) | 48 (18.5%) | ns |
| Abdominal/pelvic magnetic resonance imaging (MRI) | 54 (11.4%) | 12 (5.6%) | 42 (16.2%) | <0.001 |
| Abdominal sonography | 185 (39.1%) | 70 (32.7%) | 115 (44.4%) | 0.011 |
| Colonoscopy | 233 (49.3%) | 105 (49.1%) | 128 (49.4%) | ns |
| Fecal calprotectin measurement | 153 (32.3%) | 47 (22.0%) | 106 (40.9%) | <0.001 |

*^a^ p*-values, assigning the differences between UC and CD, were calculated using Fisher’s exact test; ns=not significant

^b^ in those 190 patients with at least one re-hospitalization

^c^ median (interquartile range)

^d^ more than one procedure per patient is possible
